# The drug tolerant persisters of *Riemerella anatipestifer* can be eradicated by a combination of two or three antibiotics

**DOI:** 10.1186/s12866-018-1303-8

**Published:** 2018-10-19

**Authors:** Tian Tang, Yanxia Wu, Hua Lin, Yongyu Li, Haojiang Zuo, Qun Gao, Chuan Wang, Xiaofang Pei

**Affiliations:** 1 Department of Public Health Laboratory Sciences, West China School of Public Health, Sichuan University, 16#, Section 3, South Renmin Road, Chengdu, Sichuan 610031 People’s Republic of China; 2 Sichuan Entry-Exit Inspection and Quarantine Bureau, Chengdu, Sichuan People’s Republic of China; 3 Institute of Preventive Veterinary Medicine, Sichuan Agricultural University, Chengdu, Sichuan People’s Republic of China

**Keywords:** *Riemerella anatipestifer*, Persisters, Antibiotics, Drug tolerance

## Abstract

**Background:**

*Riemerella anatipestifer* (RA), the causative agent of duck infectious serositis, leads to high mortality in duck flocks and great economic losses in duck industry. Previous studies on RA are largely focused on its detection, virulence factors, serology, epidemiology as well as antibiotic resistance. Neither drug tolerant persisters nor the persister level under the treatment of antibiotics has been revealed. The persisters are non-growing or dormant cells within an isogenic bacterial population; they play important roles in recurrent infection and formation of drug resistant mutants. The aim of this study is to detect the drug tolerant persisters from the exponentially grown population of RA reference strain (RA 11845) or RA clinical isolate (RA TQ3), and address whether a single antibiotic or a combination of two or three antimicrobials can eradicate the persisters at respective maximum serum/plasma concentration (C_max_).

**Result:**

With the concentration of a test antibiotic increased, a small fraction of cells in the exponentially grown culture of RA reference strain (RA 11845) or RA clinical isolate (RA TQ3) always survived, irrespective of treatment time, indicating the presence of drug tolerant presisters. A single antibiotic cannot eradicate the persisters of both RA strains at respective C_max_, except that the C_max_ of ceftiofur wiped out the population of the reference strain (RA 11845). Besides, the clinical isolate RA TQ3 presented a higher tolerance to ceftiofur in comparison to that of the reference strain (RA 11845). Combination of any two or three antimicrobials eliminated the drug tolerant persisters of RA TQ3 completely at respective C_max_.

**Conclusion:**

A sub-community of drug tolerant persisters was present in RA population. Persisters of RA TQ3 are single drug tolerant and not multidrug tolerant persisters.

**Electronic supplementary material:**

The online version of this article (10.1186/s12866-018-1303-8) contains supplementary material, which is available to authorized users.

## Background

Duck meat has traditionally been an important source of animal protein in many Asian countries; world production was approximately 4.4 million tons in 2013 [1]. Infectious diseases are therefore of increasing interest to the duck industry. *Riemerella anatipestifer* (RA), a Gram-negative, nonmotile, spore-forming, and rod-shaped bacterium, is the etiological agent of duck exudative septicemia or infectious serositis, causing great economic losses in duck industry worldwide due to high mortality (up to 75%), weight loss, and treatment cost [2–4]. Besides ducks, other poultry species such as chicken, geese, and turkeys are also susceptible to RA infection [5–8].

Previous work on RA mostly focused on its detection [9–11], virulence factors [12, 13], serology [14–16], epidemiology [17–19], and antibiotic resistance [20, 21]. Neither drug tolerant persisters nor the persister level of RA under the treatment of clinical antibiotics has been revealed. The persisters are non-growing or dormant cells within an isogenic bacterial population, capable of enduring lethal doses of antibiotics due to their inactive physiological state [22–24]. Unlike drug-resistant mutants with genetic change which can proliferate in the presence of antibiotics, persisters do not replicate in the same condition. The antibiotic tolerance of persisters is transient, non-genetic, and not inheritable [25]. Once drugs are removed, persisters restart growth and result in a population that is again antibiotic sensitive [26]. Although persister cells account for merely 0.001 to 1% of the entire bacterial population, they play important roles in recurrent infection and formation of drug resistant mutants [26]. In the present study, we sought to determine if persisters are present in RA population and address whether the maximum serum/plasma concentration (C_max_) of a single antibiotic or a combination of two or three antimicrobials, each at C_max_, can eradicate the persisters.

## Results

### Identification of persisters from exponential culture of RA

**Fig. 1:**
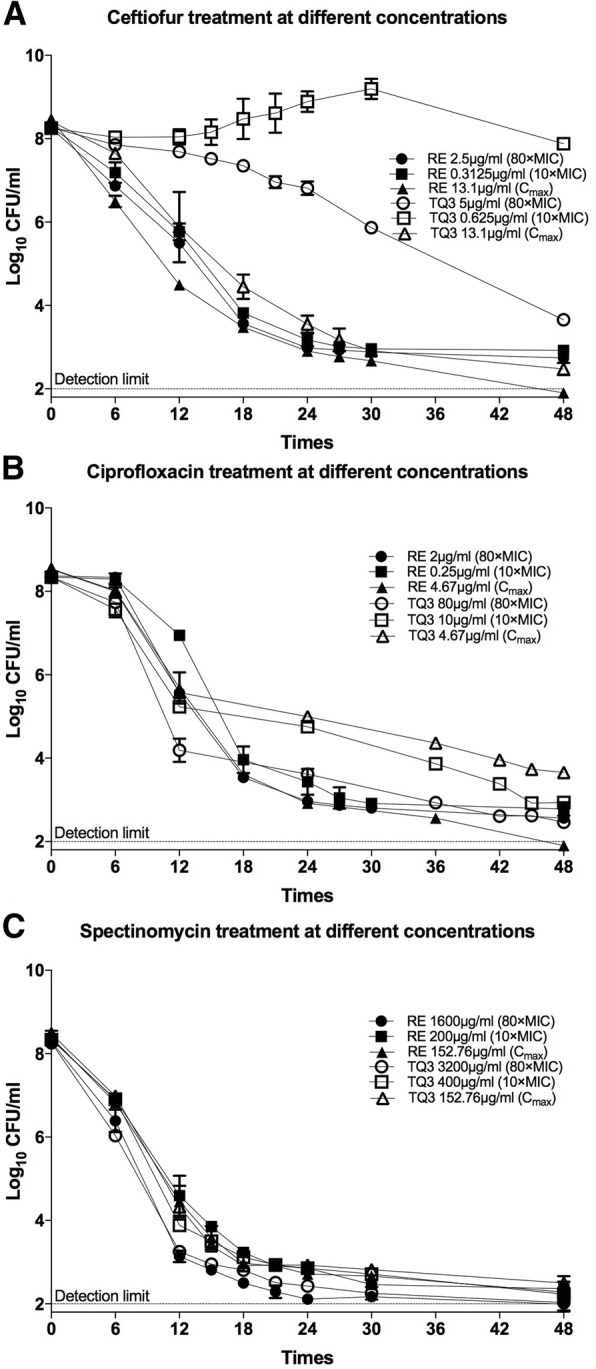
Time-dependent killing by test antibiotics alone. Exponential cells of RA 11845 and RA TQ3 were treated with different concentrations of ceftiofur (**a**), ciprofloxacin (**b**) and spectinomycin (**c**), respectively. RE presents RA reference strain, RA 11845; TQ3 presents RA clinical isolate, RA TQ3. The detection limit was 10^2^ CFU/ml. Each spot showing on the figure was the mean of three biological replicates. The error bars represent the standard deviation of the mean

Prior to investigating the drug tolerant persisters, we determined the MIC of each antibiotic for each RA strain. The results are summarized in Table 1. To detect the persisters from exponential growing culture of RA reference strain (RA 11845) or RA clinical isolate (RA TQ3), we performed a time-dependent killing experiment, using three clinical antibiotics (ceftiofur, ciprofloxacin and spectinomycin). As shown in Fig. 1a, exposure to increasing concentrations of ceftiofur resulted in a rapid decrease in bacterial population of RA 11845, with a sharp reduction > 99.9% (the number of killed cells divided by the number of cells before addition of drug) after 24 h treatment at 10- and 80-fold MIC, followed by a steady value up to 48 h, indicating the presence of ceftiofur-tolerant persisters. When ciprofloxacin was added to the culture in a final concentration of 80-fold MIC, the bacterial number barely changed within the first 6 h, then decreased drastically before reaching the plateau at 24 h (Fig. 1b). Similar biphasic killing curve was observed in the presence of 10-fold MIC of ciprofloxacin, except the time that reached the plateau was delayed to 27 h (Fig. 1b). These data suggest that ciprofloxacin-tolerant persisters existed in bacterial population of RA 11845. Spectinomycin was more efficient in killing the exponential growing cells of RA 11845 in comparison to ceftiofur and ciprofloxacin, since more than 99.9% of cells were sterilized by spectinomycin in the initial 12 h, irrespective of drug dosage (Fig. 1c). The killing curves of spectinomycin also exhibited a biphasic pattern, indicating the presence of spectinomycin-tolerant persisters.

**Table 1:** MIC of each antibiotic, C_max_ relative to MIC, and the FICI values of combining two drugs against RA 11845 or RA TQ3

Strains	MIC (μg/ml)			C_max_/MIC			FICIs					
	CEF	CIP	SPE	CEF	CIP	SPE	CEF + CIP		CEF + SPE		CIP + SPE	
RA 11845	0.03125	0.025	20.0	420	189	8	0.75	(AD)	1.0	(AD)	0.56	(AD)
RA TQ3	0.0625	1.0	40.0	210	5	4	0.75	(AD)	1.0	(AD)	1.0	(AD)

CEF = Ceftiofur; CIP = Ciprofloxacin; SPE = Spectinomycin

AD represents additive

When exponential population of RA TQ3 was challenged with ceftiofur at 80-fold MIC, the number of survivors decreased over time without showing a plateau on the killing curve (Fig. 1a). Besides, 73.2% of RA TQ3 cells were destroyed after 12 h of treatment in comparison to that of 99.8% of RA 11845 cells were abolished under a similar dosage (80-fold MIC). Collectively, this indicate that RA TQ3 are more tolerant to ceftiofur than the RA reference strain (RA 11845). Intriguingly, 10-fold MIC of ceftiofur diminished the population of RA TQ3 in the initial 6 h, but the cell number increased thereafter, indicating the generation of ceftiofur-resistant mutants which are capable of proliferating in the presence of ceftiofur (Fig. 1d). The increasing concentrations of ciprofloxacin and spectinomycin alone killed the non-tolerant cells of RA TQ3 rapidly in the initial 18 and 12 h, respectively, leaving a substantial number of survivors. The number of survivors barely changed over time, and showed a plateau on the killing curves of each drug (Fig. 1b & c), indicating that these survivors were persisters. The killing curves of each drug for each RA strains at 20- and 40-fold MIC are presented in Additional file 1: Table S1.

**Fig. 2:**
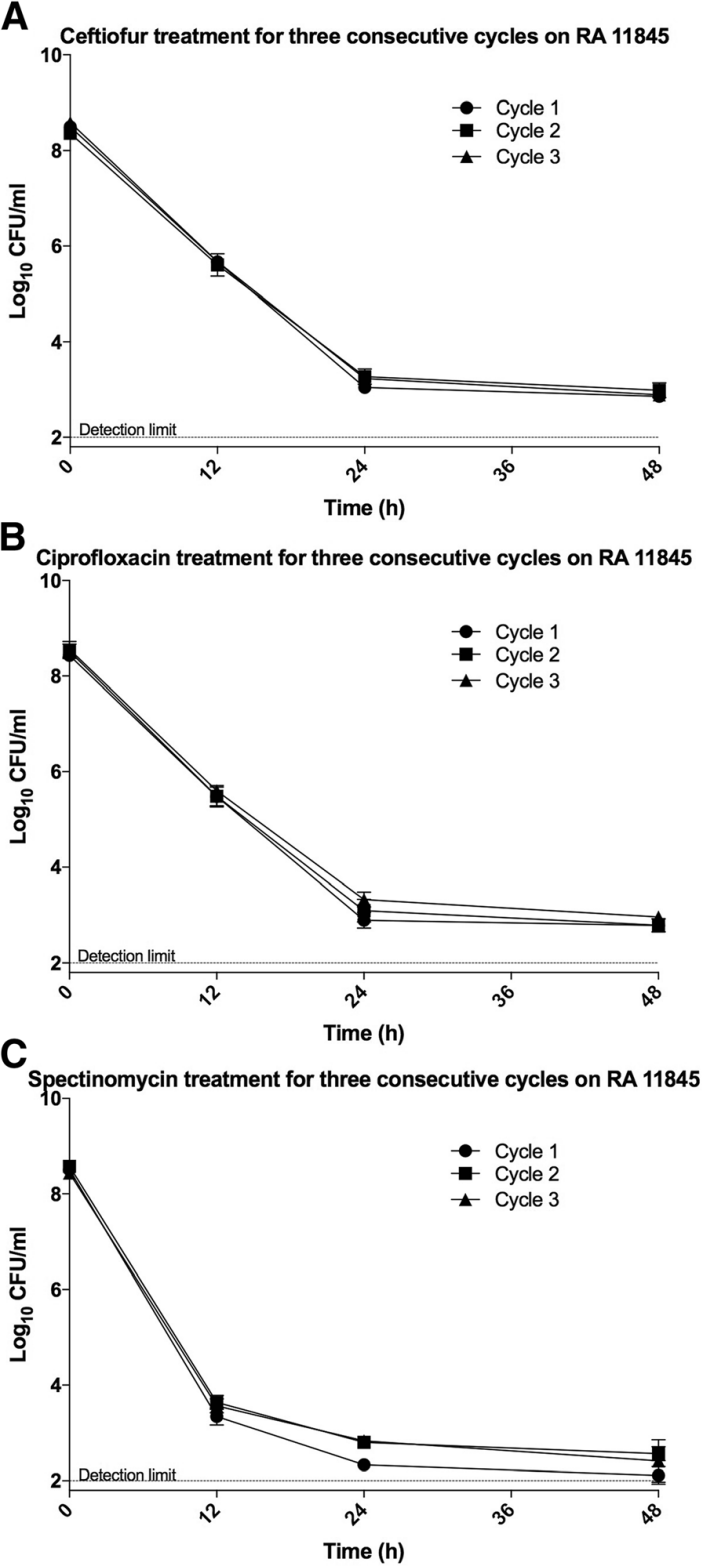
Examination for heritability of persistence. Exponential growing cells of RA 11845 were challenged with 80-fold MIC of ceftiofur (**a**), ciprofloxacin (**b**), or spectinomycin (**c**) for 48 h in three consecutive cycles. The detection limit was 10^2^ CFU/ml. Each spot showing on the figure was the mean of three biological replicates. The error bars represent the standard deviation of the mean

To further validate that the surviving bacteria were in fact persisters and not resistant bacteria, we isolated the survivors of RA 11845 from 48 h of treatment with 80-fold MIC of ceftiofur, ciprofloxacin, or spectinomycin. These survivors were regrown in fresh media to form a population in exponential phase, followed by the challenge with same antibiotic and dosage again to finish the first cycle. After three consecutive cycles, we did not observe an elevated level of survivors (Fig. 2a-c); the bacterial population derived from the survivors of each cycle was as sensitive to each antibiotic as the parental strain RA 11845. All of these evidences suggested that these survivors are persisters, because, their drug tolerance is transient and non-heritable.

### The persister level of RA under C_max_ of a single antibiotic

Given the potential of persisters to cause a relapse, it is important to address whether the C_max_ of an antibiotic can eradicate the drug tolerant persisters. As shown in Fig. 2a-c, none of the tested antibiotic was able to eliminate persister cells of RA TQ3 alone at C_max_. Similar results were observed in exponentially grown population of RA 11845, except that the C_max_ of ceftiofur wiped out all bacterial cells within a period of 48 h (Fig. 2a).

### Persisters of RA TQ3 were eradicated by a combination of two or three antibiotics

**Fig. 3:**
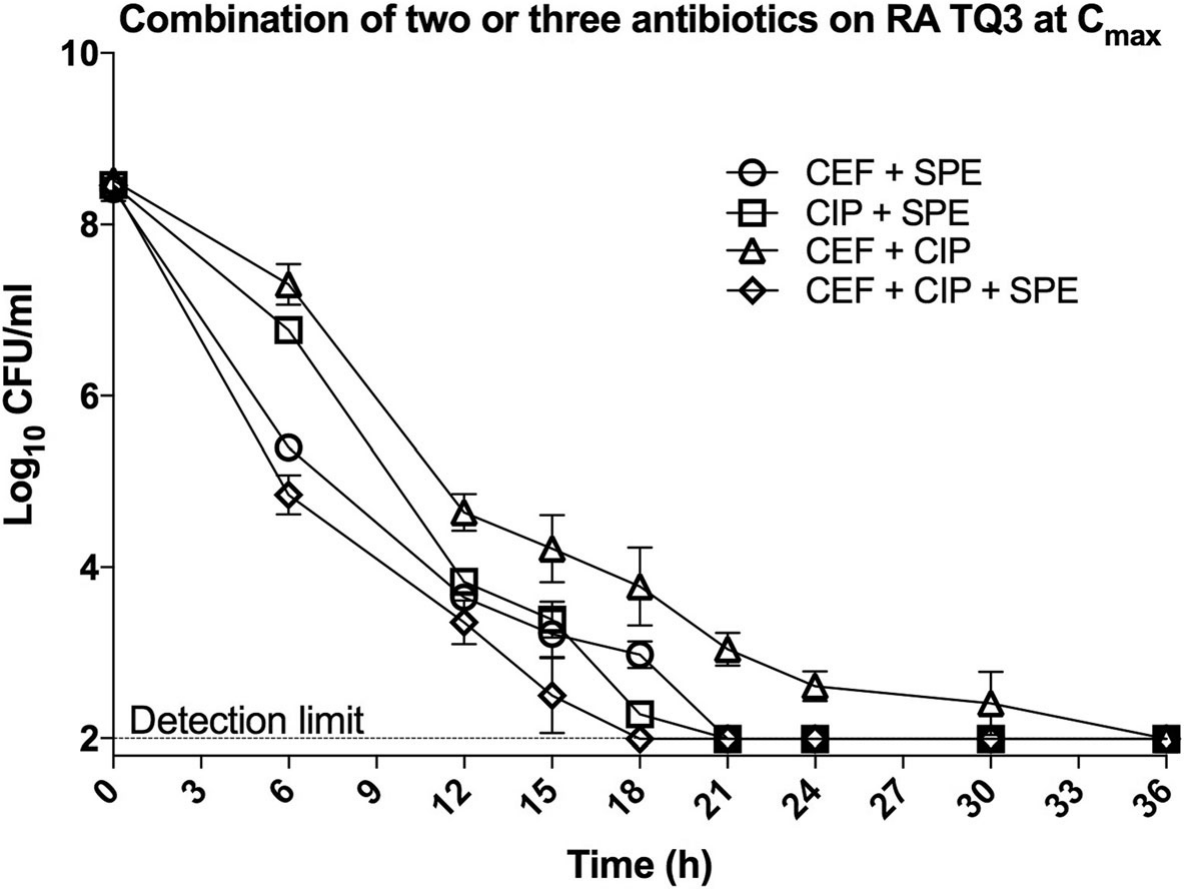
Time-dependent killing by combining two or three antibiotics at respective C_max_ against RA TQ3. The detection limit was 10^2^ CFU/ml. Each spot showing on the figure was the mean of three biological replicates. The error bars represent the standard deviation of the mean

To better understand the interactions between drugs, we performed a checkerboard assay. As shown in Table 1, the FICI values from all drug combinations for each RA strain were of > 0.5 to ≤1, indicating an additive effect between any two drugs. We therefore hypothesize that a combination of any two drugs, each at C_max_, might reduce the persister level of RA TQ3. To our surprise, spectinomycin not just killed the non-tolerant cells but eliminate the persister cells of RA TQ3 within 21 h, when used in concert with ciprofloxacin or ceftiofur. Similar results were observed when exponential growing population of RA TQ3 was challenged with drug combination of ceftiofur and ciprofloxacin, or treated with three antimicrobials simultaneously, except that the drug combination of ceftiofur and ciprofloxacin abolished the persisters of RA TQ3 within 36 h, whereas combination of all three test drugs eliminated the persisters of RA TQ3 within 18 h (Fig. 3).

## Discussion

It has been suggested that *Riemerella anatipestife* (RA) can form biofilms on certain criteria [27]. This phenomenon led us to hypothesize that persister cells might exist in RA population, because, persisters are produced in biofilms [20]. In the present study, we confirmed the presence of persister cells in RA Population. The antibiotics we used to detect persister cells from exponential growing population of RA reference strain (RA 11845) or RA clinical isolate (RA TQ3) were ceftiofur, ciprofloxacin and spectinomycin, respectively. Ceftiofur, a veterinary antibiotic, has been approved for food animal use in the United States and Europe [28]. As a third generation of cephalosporin, ceftiofur has similar bactericidal mechanism to all β-lactam antibiotics which disrupts the integrity of bacterial cell wall by inhibiting the catalytic activity of penicillin-binding proteins (PBPs) [28, 29]. Our results suggested a strong tolerance of RA TQ3 cells to ceftiofur at 80-fold MIC (Fig. 1a). This is unexpected, because, the cells of RA reference strain (RA 11845) was sensitive to ceftiofur at a similar dosage (Fig. 1a). Such robust tolerance of RA TQ3 cells to ceftiofur is probably due to its clinical origin, given that a clinical isolate of *Klebsiella pneumoniae* presented a higher tolerance to some of tested antimicrobials in comparison to the laboratory reference strain [30].

Drugs of quinolone family, such as ciprofloxacin and norfloxacin, are capable of binding to the DNA complex of type IIA topoisomerases, DNA gyrase (GyrA:GyrB) or topoisomerase IV (topo IV) (ParC:ParE), resulting in double-strand DNA breaking and cell death [31, 32]. In vitro assessment of a collection of quinolones against a dozen of veterinary pathogens indicated that ciprofloxacin was the most active quinolone against *Mycoplasma hyopneumoniae*, *Bordetella bronchiseptica*, *Pasteurella multocida*, and *Haemophilus pleuropneumoniae* [33]. Our data suggested that the bactericidal effect of ciprofloxacin on RA was poor in the first few hours; the cell number of RA TQ3 dropped slowly, irrespective of dosage. Such phenomenon was more evident on RA 11845 where the cell number hardly changed during the initial 6 h of exposure (Fig. 1b). This “delayed killing effect” of ciprofloxacin was also presented in certain strains of *Klebsiella pneumoniae* and *Yersinia pestis* [34, 35]. But the actual cause remains to be elucidated.

Spectinomycin has been traditionally defined as a bacteriostatic agent, working by destabilizing the binding of peptidyl-tRNA to ribosome [29, 36]. In this study, spectinomycin destroyed 99.9% of cells within 12 h when applied to exponential growing culture of RA 11845 or RA TQ3 (Fig. 1c). Such bactericidal action of spectinomycin was not limited to RA, but on *Neisseria gonorrhoeae* as well [37]. It was demonstrated that spectinomycin showed a higher binding affinity to the 30S ribosomal proteins of *Neisseria gonorrhoeae* than that to those of *E coli* where it presented a bacteriostatic activity [38]. Perhaps, a similar high avidity of spectinomycin to the 30S ribosomal proteins of RA might exist which led to the bactericidal action.

As C_max_ representing the highest concentration of a drug that can reach in serum/plasma after a single dose, we sought to address whether a test antibiotic can abolish all cells of RA at C_max_. Our data suggested that the C_max_ of ceftiofur wiped out the non-tolerant cells of RA TQ3 rapidly and left a steady population of persister cells (Fig. 1a). This is unanticipated, because a concentration of ceftiofur at 80-fold MIC was unable to eradicate even the non-tolerant cells of RA TQ3 (Fig. 1a). It appears that the non-tolerant cells of RA TQ3 can endure a degree of ceftiofur, but succumbs to a high concentration of ceftiofur at C_max_ (13.1 μg/ml, 210-fold MIC, Table 1). In contrast, the C_max_ of ceftiofur eliminated all bacterial cells of RA 11845 within 48 h of treatment. We attributed this to the following reasons: the high concentration of ceftiofur at C_max_ (13.1 μg/ml, 420-fold MIC, Table 1) improved its own bactericidal activity; the reference strain (RA 11845) was more vulnerable to ceftiofur, given that 10- and 80-fold MIC of ceftiofur killed the sensitive cells of RA 11845 within 24 h but failed to eliminate the non-tolerant cells of RA TQ3 during the same period of time (Fig. 1a). Clinically, the concentration of a drug was fluctuated in serum/plasma. Therefore, the true effect of an antimicrobial was anticipated to be lower than that at a constant C_max_. Moreover, we cannot expect to destroy persisters by increasing the dosage of a drug progressively. Because, with an increased serving dosage, the toxicity of a drug also increased. As different antibiotics that have different modes of action, we wondered whether combining two or three antimicrobials could abolish the RA persisters at C_max_, since several reports has demonstrated that drug combination could impair or even eradicate the persisters [39–41]. Our data indicated that the persisters of RA TQ3 were eliminated completely by a combination of any two or three drugs. In a previous study, Ramandeep and colleagues [39] have revealed two types of persisters: one-drug tolerant persisters, and multidrug tolerant persisters. The one-drug tolerant persisters are capable of enduring one drug but are not cross-tolerant to others drugs. The multidrug tolerant persisters are able to endure multiple antimicrobials. Apparently, the RA persisters are one-drug tolerant persisters and not multidrug tolerant persisters, because they cannot survive in the presence of multiple antibiotics.

The host environment is more complicated than in vitro conditions. Except the fluctuation of drug concentration in serum/plasma that we mentioned above, other factors such as the host-produced substances and local pH also affect the action of an antibiotic. Therefore, we cannot conclude that drug combination is more effectiveness against RA persisters comparing to a single antimicrobial in vivo. However, this study presents a potential solution to eradicate persisters, not only for RA, but for other zoonotic pathogens.

## Conclusion

In this study, we confirmed the presence of drug tolerant persisters in RA population. The test antibiotics, including ceftiofur, ciprofloxacin and spectinomycin, cannot eradicate the persisters of RA TQ3 alone at C_max_. However, a combination of two or three antibiotics eliminated the persisters of RA TQ3 completely at C_max_. Our investigations provide a way to wipe out the drug tolerant persisters, which is helpful for the clinical treatment of RA infection in poultry.

## Methods

### Antibiotics, bacterial strains and culture conditions

Ceftiofur hydrochloride was purchased from Aladdin (Shanghai, China). Ciprofloxacin hydrochloride and spectinomycin were obtained from Sigma-Aldrich (St. Louis, MO, USA). All antibiotics used were of analytical standard (purity > 99.9%). A reference strain, RA 11845, was purchased from American Type Culture Collection (ATCC). A clinical isolate, RA TQ3, was a kind gift from Dr. Lin at Sichuan Entry-Exit Inspection and Quarantine Bureau. The RA TQ3, sensitive to all test antimicrobials, was isolated from a batch of imported breeding chickens in 2016. Strains were routinely grown on trypticase soy agar (TSA) with 5% sheep blood. Due to the growth deficiency of RA in Mueller-Hinton broth (MHB), trypticase soy broth (TSB) was used to culture cells and determine the MICs of antibiotics.

### The MIC measurement

The MIC measurement was conducted as described previously [42]. In brief, all wells in each row of a 96-well plate (Costar 3599, Corning, New York, USA) were filled with 100 μl of TSB medium, except the first one, which was filled with 200 μl of TSB medium to serve as a blank control. 100 μl of fresh medium containing 32-fold the expected MIC of an antimicrobial agent was then added to the second well, followed by a 2-fold serial dilution from the second well to the 11th well. The last well contained no drugs was served as a positive control of growth. Overnight culture of RA 11845 or RA TQ3 was inoculated in 1 ml of fresh TSB in a ratio of 1:10, and incubated at 37 °C with shaking at 180 rev/min. When the optical density (OD) reached at ~ 0.2 (10^9^ CFU/ml), 100 μl culture was diluted at 1:1000 in fresh TSB medium. 100 μl of the diluted culture containing 10^5^ CFU/ml of cells were inoculated from the second well to the 12th well. The plate was capped and incubated at 37 °C for 18 h, OD was then measured by a Multiskan GO Spectrophotometer (ThermoFisher Scientific, San Jose, CA, USA). The MIC was determined as the lowest concentration of a respective antibiotic which inhibited the growth of cells. The MIC measurement for each drug was repeated three times.

### Checkerboard assay

The interactions between antibiotics were evaluated by the checkerboard assay [43, 44]. The concentration range of each antibiotic in each drug combination was from 2-fold to 1/16-fold the MIC. The turbidity was checked after 18 h of incubation at 37 °C. The fractional inhibitory concentration index (FICI) was calculated using the following formula: FICI = MIC_A + B_ / MIC_A_+ MIC_B + A_ /MIC_B_. The MIC_A_ and MIC_B_ represent the MIC of drug A and drug B alone, respectively. The MIC_A + B_ is the MIC of drug A in the presence of drug B, and vice versa for MIC_B + A_. The FICI values were interpreted as follows: synergy, FICI ≤0.5; additivity, FICI > 0.5 to ≤1; no interaction, FICI > 1 to ≤4; antagonism, FICI > 4. For each drug combination, the checkerboard assay was repeated three times.

### Determination of persister level

The persister level of each RA strain was evaluated by a time-dependent killing experiment, using three antibiotics (ceftiofur, ciprofloxacin, and spectinomycin). Briefly, 100 μl overnight culture of RA 11845 (~ 4.5 × 10^9^ CFU/ml) or RA TQ3 (~ 3.8 × 10^9^ CFU/ml) was inoculated in 100 ml (1:1000) of fresh TSB medium, and incubated at 37 °C with shaking at 180 rev/min. The culture was divided into aliquots of 25 ml when an OD_600_ value arrived at 0.1 ~ 0.2 (1.7 × 10^8^ ~ 3.8 × 10^8^ CFU/ml). A single antibiotic was added to the aliquots of culture in a final concentration of 10-, 20-, 40- and 80-fold the MIC, respectively. Cultures were incubated as described above. At designated time points, 1 ml sample from each culture was withdrawn, washed and resuspended in an equal volume of 1.0% (*w*/*v*) saline solution. Cell suspension was then submitted to bacterial numeration using standard plate counting method. The persister level was determined by the number of survivors on the plateau of the biphasic killing curves. All experiments were performed with three independent biological replicates.

In clinical practice, the serum/plasma concentration of an antimicrobial reaches the peak (C_max_) sometime post administration. Therefore, it is of great importance to evaluate the persister level of each RA strain under the treatment of a single antibiotic at C_max_ due to the potential of persisters to cause a relapse. For this purpose, we performed a similar time-dependent killing experiment as mentioned above, except that the final concentration of a drug in culture was adjusted to C_max_. Due to the shortage of pharmacokinetic parameters of ciprofloxacin and spectinomycin in ducks, the C_max_ of these antibiotics for chickens are adopted instead. The C_max_ values of all tested antibiotics are listed in Table 2.

**Table 2:** The peak serum/plasma concentration (C_max_) of antimicrobial agents

Antibiotics	Route of administration	Avian species	C_max_ (μg/ml)	Reference
Ceftiofur	Subcutaneous injection	American black ducks	13.1	[45]
Ciprofloxacin	Oral administration	broiler chickens	4.67	[46]
Spectinomycin	Intramuscular injection	Broiler chickens	152.76	[47]

### Inheritability of drug tolerance for persisters

An aliquot of exponential phase culture of RA 11845 was exposed to 80-fold MIC of ceftiofur, ciprofloxacin, or spectinomycin for 48 h. At designated time points, cells were harvested, washed, and counted on non-selective agar (TSA with 5% sheep blood). At the end of each treatment, 100 μl culture was withdrawn, washed and inoculated in 10 ml of fresh TSB medium. After incubated at 37 °C overnight with shaking at 180 rev/min, cells were diluted at 1:1000 in fresh TSB medium and regrew to exponential phase to complete the first cycle. Cells were then challenged with antibiotics as described above, followed by cell numeration at designated time points. The procedure was repeated for three consecutive cycles.

### The persister level of RA TQ3 under the treatment of a combination of two or three drugs

A batch of exponential phase culture of RA TQ3 was challenged with a combination of two or three antimicrobials. The final concentration of each antibiotic in cell culture was adjusted to C_max_. At designated time points, culture samples were taken, washed, and plated after serial dilution for CFUs. The level of persisters was again evaluated by the number of survivors showing on the plateau of the killing curves. For each drug combination, the experiment was repeated three times.

## Additional file

Additional file 1: **Table S1.** Exponentially grown RA population was exposed to 20- and 40-fold the MIC of ceftiofur (A), ciprofloxacin (B) and spectinomycin (C), respectively. RE presents RA reference strain, RA 11845; TQ3 presents RA clinical isolate, RA TQ3. The detection limit was 10^2^ CFU/ml. Each spot showing on the figure was the mean of three biological replicates. The error bars represent the standard deviation of the mean. (TIF 940 kb)

## Abbreviations

CEF: Ceftiofur
CFU: Colony-forming unit
CIP: Ciprofloxacin
C_max_: Maximum serum/plasma concentration
FICI: Fractional inhibitory concentration index
MHB: Mueller-Hinton broth
MIC: Minimum inhibitory concentration
OD600: Optical density at 600 nm
RA: *Riemerella anatipestifer*
SPE: Spectinomycin
TSA: Trypticase soy Agar
TSB: Trypticase soy broth
