# Supplementary figures and images for: The drug tolerant persisters of Riemerella anatipestifer can be eradicated by a combination of two or three antibiotics
Source: BMC Microbiol. 2018 Oct 19;18:137. doi: 10.1186/s12866-018-1303-8 (PMC6194556; doi:10.1186/s12866-018-1303-8)

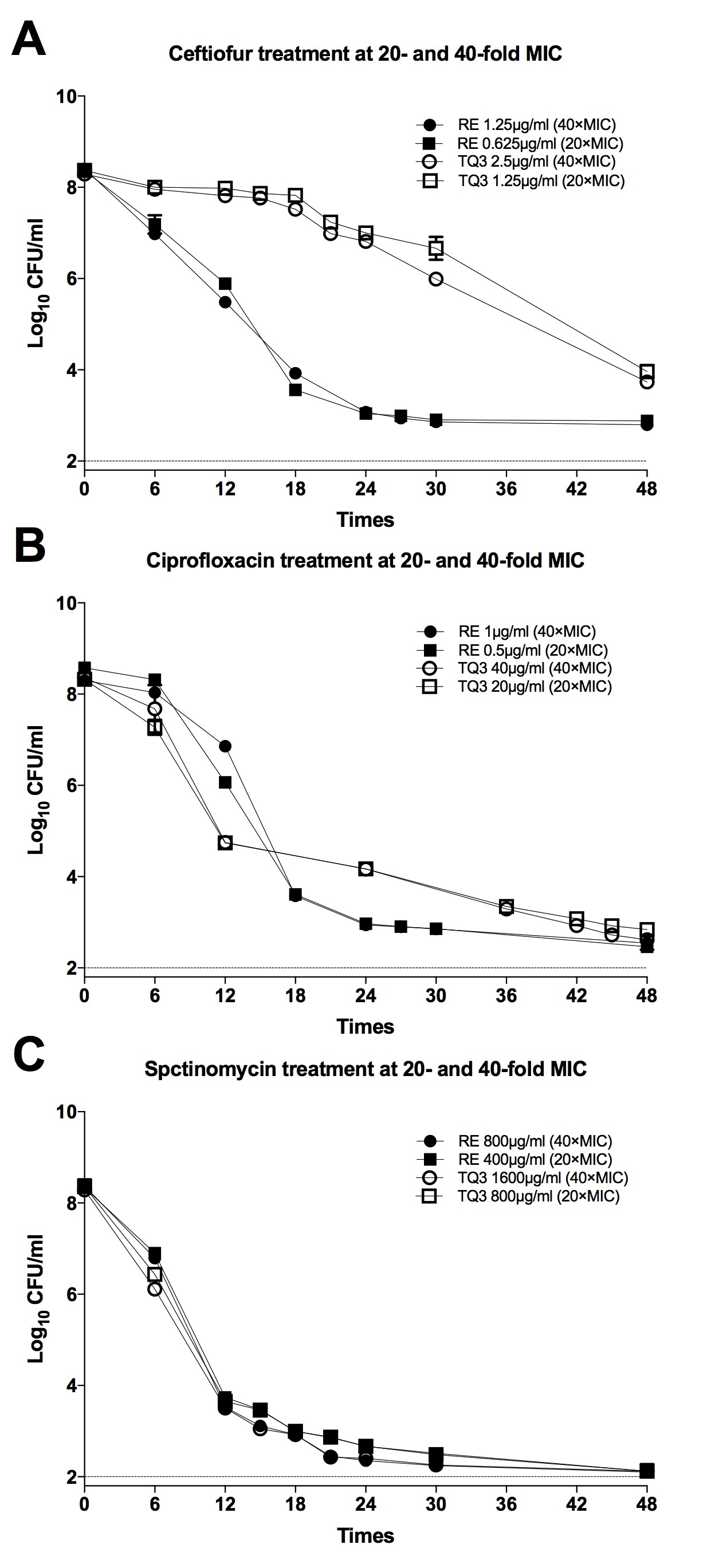

Supplement: Supplementary file 1 — Table S1. Exponentially grown RA population was exposed to 20- and 40-fold the MIC of ceftiofur (A), ciprofloxacin (B) and spectinomycin (C), respectively. RE presents RA reference strain, RA 11845; TQ3 presents RA clinical isolate, RA TQ3. The detection limit was 102 CFU/ml. Each spot showing on the figure was the mean of three biological replicates. The error bars represent the standard deviation of the mean. (TIF 940 kb) [file 12866_2018_1303_MOESM1_ESM.tif]
